# From plankton to fish: 21st-century redistribution of marine biodiversity and the changing role of rare species

**DOI:** 10.1126/sciadv.aee7253

**Published:** 2026-10-07

**Authors:** Elena Couce, Lily Greig, Georg H. Engelhard, John K. Pinnegar, Keith M. Cooper, Pierre Hélaouët, Laurene Pecuchet, Myron A. Peck, Martin Lindegren, Murray S. A. Thompson

**Affiliations:** ^1^ Centre for Environment, Fisheries and Aquaculture Science (Cefas), Lowestoft Laboratory, Pakefield Road, Lowestoft, Suffolk NR33 0HT, UK.; ^2^ School of Environmental Sciences, University of East Anglia (UEA), Norwich NR4 7TJ, UK.; ^3^ The Marine Biological Association (MBA) The Laboratory, Citadel Hill Plymouth, Devon PL1 2PB, UK.; ^4^ Norwegian College of Fishery Science, UiT, The Arctic University of Norway, Tromsø, Norway.; ^5^ Royal Netherlands Institute for Sea Research (NIOZ), Landsdiep 4, 1797 SZ ‘t Horntje (Texel), Netherlands.; ^6^ DTU Aqua, National Institute of Aquatic Resources, Kongens Lyngby, Denmark.

## Abstract

Predictions of climate-driven marine biodiversity change typically overlook noncommercial and rare taxa, leading to an incomplete and potentially biased understanding of change. We address this gap by quantifying α-, β-, and γ-diversity using Hill numbers consistently across co-occurring phytoplankton, zooplankton, benthos, and fish taxonomic groups. Using Bayesian additive regression trees and extensive survey data, we project climate-driven biodiversity change in the northeast Atlantic until 2100. We show that climate change could redistribute biodiversity both within taxonomic groups, altering the contribution of rare species, and across the food web. Widespread increases in fish and benthos species richness, reflecting poleward migration, often coincided with declines in zooplankton and phytoplankton richness, potentially disrupting energy transfer from lower to higher trophic levels. By identifying areas with high biodiversity across taxonomic groups and predicting how these could change in the future, we provide a framework to support proactive conservation actions to help achieve global biodiversity targets in a changing climate.

## INTRODUCTION

Global biodiversity is undergoing rapid transformation under intensifying human pressures and climate change (*1*) with marine ecosystems facing particularly acute risks (*2*, *3*). These biodiversity changes have undermined essential ecosystem functions and services for human well-being (*1*, *4*–*6*) with projected trends that could jeopardize global food security (*7*, *8*). Despite this wealth of evidence of climate-driven changes in marine biodiversity, little is known about how responses differ across co-occurring taxonomic groups, e.g., phytoplankton, zooplankton, benthos, and fish. This is partly due to the scarcity of high-quality biodiversity data (*9*) and biases caused by substantial differences in the spatial and temporal distribution of survey data across different taxonomic groups (*10*). Large-scale assessments, therefore, typically either combine biodiversity trends from multiple taxonomic groups into a single overall estimate (*2*, *3*, *11*) or focus on change within a taxonomic group (*12*–*14*), often relying on indicator species, such as taxa of commercial interest or conservation concern (*15*–*17*). Biodiversity assessments need to go beyond indicator species to gauge a more complete picture of change that can be multidimensional and scale dependent (*18*–*20*). Thus, substantial knowledge gaps remain in understanding past, present, and future climate change effects on biodiversity within and between taxonomic groups because the contribution of many species has not been quantified.

Among the species whose contributions remain poorly quantified, rare species are particularly overlooked because their distributions are poorly understood [(*15*, *16*), but see (*17*) for distribution models of rarer taxa], yet they contribute disproportionately to species richness (*21*–*23*) and play crucial roles in ecosystems. For example, they can enhance ecosystem stability and resilience by contributing distinct trait combinations (*24*, *25*) and by providing weak links in food webs (*26*–*28*). Determining how biodiversity is changing within and between taxonomic groups in a systematic way, including the contributions of rare species, is critical because shifts in the distribution of diversity in food webs, from resources to consumers, constrain nutrient uptake and the efficiency of biomass production (*5*, *29*) and because species-rich assemblages can better maintain ecosystem functioning under environmental change (*30*).

Rarefaction and extrapolation based on Hill numbers provide a unified framework to quantify biodiversity across multiple dimensions and spatial scales (*31*, *32*). This includes α-diversity (sample-level species richness), β-diversity (regional differences in species composition among samples), and γ-diversity (regional species richness). Comparing Hill numbers of order 0 and 2, which estimate total species richness and the number of dominant species, respectively, allows us to quantify the relative contribution of rare species. Here, we conduct the first systematic, multidimensional assessment of biodiversity change across four co-occurring marine taxonomic groups in the Northeast Atlantic: phytoplankton, zooplankton, benthos (i.e., seafloor-inhabiting macroinvertebrates), and fish. Using extensive long-term survey data, we reconstruct past and present biodiversity patterns for each taxonomic group and train Bayesian additive regression trees (BART) models to project changes from 1993 to 2100.

In temperate marine regions such as the Northeast Atlantic, warming is expected to increase γ-diversity over the next decades when gains of warm-affinity species from more species-rich lower latitudes exceed losses of cold-affinity species retreating poleward (*33*, *34*). The rate and magnitude of these changes are likely to differ among taxonomic groups because of contrasts in mobility, life-history traits, dispersal capacity, and sensitivity to environmental change (*35*, *36*). In addition to changes in overall diversity, climate-driven species turnover is expected to alter species dominance structure by affecting the balance between rare and common species (e.g., previously rare warm-affinity species can be expected to expand their range, while some formerly common cold-affinity species ranges may decline). We therefore expected climate change (i) to increase γ-diversity across taxonomic groups, but with (ii) differences in the rate and magnitude of change among taxonomic groups, and (iii) to observe shifts in species dominance structure. By developing predictive biodiversity indicators that capture climate-driven change across multiple dimensions and spatial scales, we provide an integrated framework to inform conservation strategies and guide progress toward international biodiversity targets under shifting baselines.

## RESULTS

Here, we present results for the Shared Socioeconomic Pathway 2-4.5 (SSP2-4.5), a mid-range greenhouse gas concentration trajectory. Projections for additional warming scenarios (SSP1-2.6 and SSP5-8.5) are provided in the Supplementary Materials.

### Climate-driven biodiversity change across taxonomic groups (Hill number 0)

Our model projections under SSP2-4.5 reveal divergent directions of γ-diversity change across taxonomic groups, contradicting our first hypothesis. Specifically, γ-diversity is projected to increase widely for fish and benthos, decrease for zooplankton, and show contrasting regional patterns of change for phytoplankton (Fig. 1, Table 1, and fig. S2). For fish and benthos, increases were consistent across scales and scenarios, with widespread gains in γ- and β-diversity and largely positive changes in α-diversity, although benthic α-diversity showed localized declines (figs. S1 to S4). Zooplankton exhibited widespread and consistent decreases in α-diversity in the continental shelf and contrasting regional patterns of change in β-diversity (figs. S3 and S4). Phytoplankton α-diversity declined across most of the region but increased along the southern UK, Ireland, and French coastal areas, with the area of increase expanding under stronger warming until nearly ubiquitous under SSP5-8.5 (fig. S3). β-Diversity for phytoplankton predominantly decreased, with scattered increases, indicating a tendency toward homogenization of species composition (fig. S4). For both phytoplankton and zooplankton α- and β-diversity, model fits were low (Table 1). For models of γ-diversity and Hill number 0, climate-related variables (temperature, salinity, oxygen, and their variability) consistently explained more variation than static habitat predictors such as substrate composition (fig. S6).

**Fig. 1.**
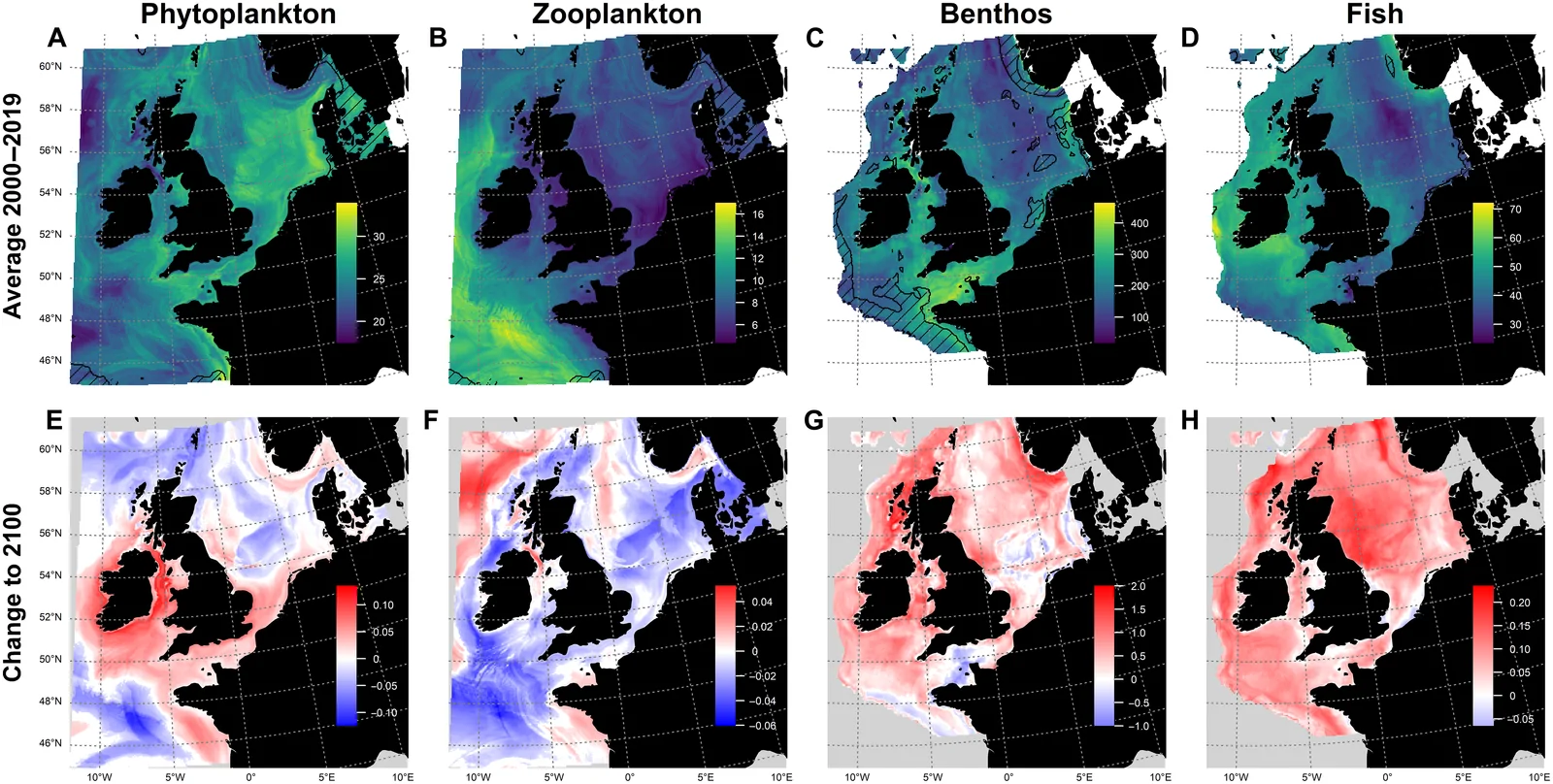
γ-Diversity patterns and trends. Spatial patterns in γ-diversity Hill number 0 (**A** to **D**) and its temporal change (**E** to **H**) for phytoplankton (A and E), zooplankton (B and F), benthos (C and G), and fish (D and H). Spatial patterns were calculated as means for the reference period of 2000–2019, with hashed regions indicating where environmental variables were outside the models’ training range. Annual change (Sen’s slopes) was estimated for the period 1993–2100 under SSP2-4.5. Red and blue cells are areas having significant (*P* < 0.05) increasing and decreasing trends, respectively, while white cells are areas with nonsignificant change. Projections for benthos and fish cover a smaller area compared to those for plankton because the former were constrained to regions with data on seabed substrate (i.e., a predictor in their models).

**Table 1.** Explained variance in BART models. *R*^2^ values for BART models showing the proportion of variance in each response explained by the predictors, averaged over the posterior draws.

Diversity	Hill number	Phytoplankton	Zooplankton	Benthos	Fish
γ	0	0.14	0.41	0.68	0.63
γ	2	0.16	0.45	0.69	0.85
α	0	0.02	0.07	0.47	0.39
β	0	0.01	0.23	0.56	0.44

Summed across the four taxonomic groups, widespread increases in γ-diversity are projected (Fig. 2). While differences in sampling, analytical methods, and taxonomic resolution among taxonomic groups mean that absolute species numbers are not directly comparable, normalizing each group’s γ-diversity and weighting them equally allows us to derive a composite indicator of broad ecosystem-level change, while avoiding dominance by highly speciose taxonomic groups such as benthos. This integrative view highlights where overall biodiversity is projected to increase or decline and complements the group-specific results by indicating regions where changes are consistent or divergent across trophic levels—information that can help identify potential conservation priority areas.

**Fig. 2.**
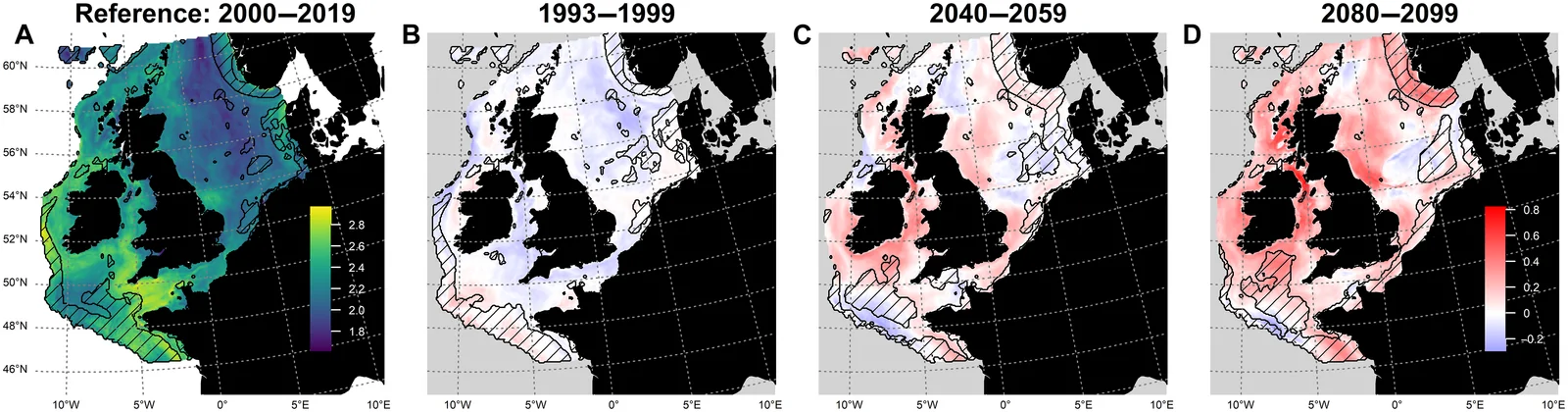
Total relative γ-diversity across taxonomic groups. Relative γ-diversity (Hill number 0) summed across phytoplankton, zooplankton, benthos, and fish (equal weighting after normalizing group-specific estimates) for (**A**) the reference period 2000–2019. Change in total relative γ-diversity compared to the reference period for (**B**) 1993–1999, (**C**) 2040–2059, and (**D**) 2080–2100 based on SSP2-4.5. Hashed areas show regions where projected environmental variables were outside any of the models’ training ranges. This composite indicator highlights areas where biodiversity increases or declines consistently across taxonomic groups.

### Differences in rates of change among taxonomic groups (Hill number 0)

Our projections confirm our second hypothesis, showing clear differences in the rates of temporal biodiversity change among taxonomic groups. Under SSP2-4.5, benthos γ-diversity increased by ∼45 species and fish by ∼10 species over 1993–2099, both equivalent to ∼0.2% per year on average (Fig. 1, G and H, and fig. S1, K and L). Phytoplankton exhibited an initial decline, followed by a subsequent increase, resulting in little net change over the century, while zooplankton declined slightly, losing ∼1 species or ∼ 0.1% per year (Fig. 1, E and F, and fig. S1, I and J).

### Rare species contribution to biodiversity (Hill numbers 0 and 2)

Projected patterns of change in the proportion of rare species to γ-diversity reveal complex, taxon-specific, and region-specific responses to climate change, highlighting the variable role of rare species in driving biodiversity change. The magnitude and direction of the changes varied markedly across groups and regions (Fig. 3 and figs. S1, M to P, and S5).

**Fig. 3.**
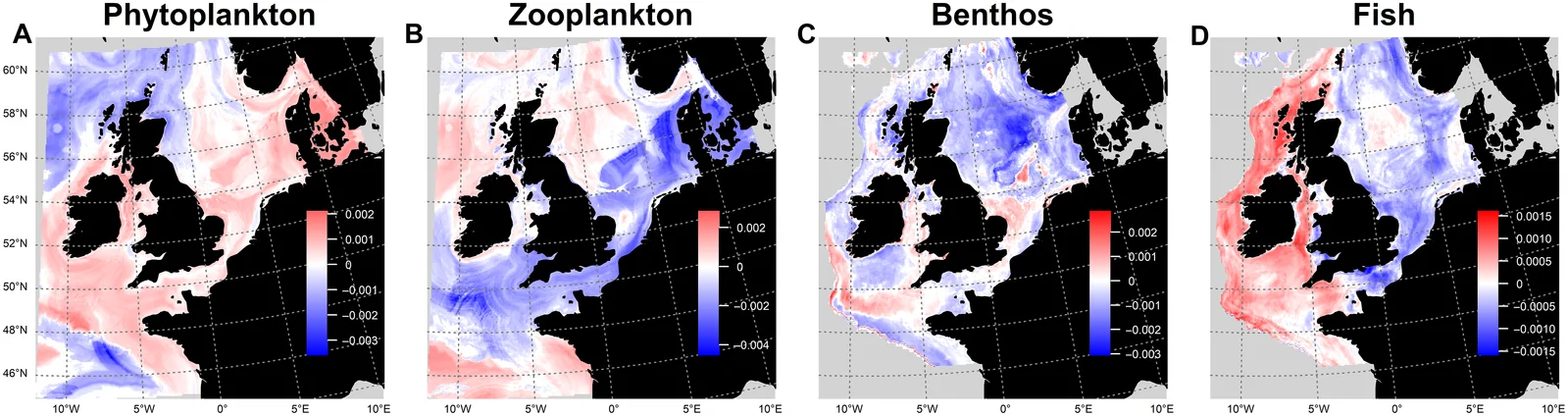
Trends in proportion of rare species. Annual change (Sen’s slopes) in the proportions of rare species for γ-diversity in (**A**) phytoplankton, (**B**) zooplankton, (**C**) benthos, and (**D**) fish for the period 1993–2100 under SSP2-4.5. Red and blue cells show significant (*P* < 0.05) increasing and decreasing annual change, respectively, with white cells showing areas with non-significant change.

For benthos, the proportion of rare species was projected to decline across most of the domain (Fig. 3C and fig. S5, C, G, and K), which, when considered alongside widespread increases in richness (Fig. 1G and fig. S2, C, G, and K), reveals range expansion of previously rare species. For fish, a similar decline in the proportion of rare species was projected across the North Sea, reflecting range expansion, but in the western region, the proportion of rare species increased, driven instead by the arrival of new rare species (Fig. 3D and fig. S5, D, H, and L). For zooplankton, concurrent declines in the proportion of rare species and γ-diversity in the southern North Sea and Celtic Sea suggest biodiversity loss primarily through extirpation of rare species (Fig. 3B and fig. S5, B, F, and J). By contrast, simultaneous increases in the proportion of rare zooplankton species and γ-diversity across much of the higher latitude areas of the study region and moving west from the Bay of Biscay highlight biodiversity change driven by the addition of new rare species. For phytoplankton, declines in both the proportion of rare species and γ-diversity coincided in the northern North Sea and across northern and southwestern regions, signaling the loss of rarer taxa, while in the northwest, increases in both metrics indicated biodiversity change driven by the arrival of rare phytoplankton species (Fig. 3A and fig. S5, A, E, and I). Similar to the models of Hill number 0, climate-related variables generally explained more variation than static habitat predictors for models of Hill number 2 (fig. S7).

## DISCUSSION

Our multidimensional assessment of biodiversity projections across co-occurring marine taxonomic groups revealed divergent future trajectories under climate change. Consistent with our first hypothesis, fish and benthos showed widespread increases in γ-diversity in the study area. These gains align with previous evidence of poleward biodiversity increases in temperate marine systems, where warm-affinity species expand into higher latitudes (*37*, *38*). In contrast, zooplankton and phytoplankton did not exhibit comparable increases, highlighting that climate-driven gains in γ-diversity are not uniform across the marine food web. Changes in the proportion of rare species were central to climate-driven marine biodiversity change, yet these dynamics were more complex than a simple poleward influx of species from the south. Crucially, assessing changes in the proportion of rare species can reveal biodiversity shifts not evident from species richness or composition alone, offering early warning of change before extirpations and supporting the development of indicators that enable preventive rather than reactive policy interventions (*39*, *40*).

By projecting emergent biodiversity patterns directly, explicitly incorporating rare species, and quantifying uncertainty, our framework extends and complements conventional and emerging species-distribution modeling approaches that focus on individual species or subsets of taxa (*41*–*43*). Species-distribution models remain essential for identifying range shifts, habitat gains and losses, and taxa requiring targeted conservation or management, but scaling them across diverse assemblages requires species-level data and model calibration for many taxa, including rare and poorly sampled species. Our approach instead models biodiversity variables directly, allowing changes in α-, β-, and γ-diversity, dominance structure, and rare-species contributions to be assessed consistently across major food-web components. This provides scalable indicators of assemblage reorganization and potential early warning signals, particularly when the question concerns emergent properties such as diversity, rarity, or abundance structure. By focusing on emergent properties of assemblages, aggregate-variable models reveal ecosystem-scale biodiversity change that may not be apparent from species-level information alone. Species-level analyses provide a complementary line of evidence, helping to uncover key taxa and processes responsible for these broader patterns.

Our projections reveal divergent responses to climate change across the marine food web. As anticipated in our second hypothesis and consistent with known differences in dispersal capacity, life-history traits, and thermal sensitivity (*35*, *36*), taxonomic groups responded at largely distinct rates and magnitudes. These imbalances could alter ecosystem structure and energy flow (*7*, *8*). In particular, declines in prey diversity (e.g., zooplankton) may weaken the “portfolio effect,” whereby a diverse prey base stabilizes energy transfer to higher trophic levels (*44*–*46*). If biodiversity gains occur primarily among predators, such as piscivorous fish in the Northeast Atlantic (*47*), then this decoupling between trophic levels could destabilize food webs and ultimately threaten food security in fisheries that depend on primary and secondary production in plankton (*48*).

Climate-driven shifts in the dominance structure of marine assemblages further support our third hypothesis. We found contrasting patterns of change across taxonomic groups and regions, indicating that processes more complex than simple range expansion of warm-affinity species were driving these changes. These changes may precede, and at times predict, subsequent shifts in species richness, underscoring the value of rarity-based indicators in detecting emerging ecological transitions. More broadly, our results align with a long ecological tradition recognizing aggregate properties, such as species abundance distributions, size spectra, and commonness-rarity patterns, as informative descriptors of community structure (*22*, *49*, *50*). These emergent patterns integrate many demographic and ecological processes and can reveal ecosystem reorganization even when species-level responses are heterogeneous or uncertain. Tracking the proportion of rare species therefore provides a complementary indicator of climate-driven community change, distinguishing biodiversity gains driven by newly arriving rare species from losses of rare taxa or expansions of formerly rare species (*51*).

Marine conservation strategies that integrate predictive indicators capable of detecting contrasting food-web responses will be essential to sustaining ecosystem functions, ensuring food security, and achieving international biodiversity targets under climate change. Future work could refine our approach by integrating additional marine ecosystem components, improving climate and human-pressure projections, and extending the framework beyond taxonomic groups. For example, the same approach could be used to deconstruct biodiversity along functional axes such as diet, trophic position, metabolism, dispersal capacity, and body size. In marine systems, body size and metabolism are particularly relevant because they mediate energy demand, trophic interactions, and vulnerability to warming, while diet and trophic position determine how climate-driven changes propagate through food webs (*52*). Applying aggregate-variable projections to functional diversity and functional rarity could therefore strengthen links between biodiversity indicators and the ecosystem processes they are intended to represent, especially where rare species carry distinctive trait combinations that support vulnerable functions (*25*, *53*). Such a framework could also be applied to terrestrial and freshwater ecosystems, which experience different dispersal constraints and different human pressures, to test whether similar patterns to those reported here exist across realms.

Our findings offer a framework to assess progress toward biodiversity targets under shifting baselines, supporting global and regional policy frameworks for biodiversity protection and conservation. We provide leading indicators that can inform preventive action, rather than those that simply document change once it has occurred (*40*). Embedding these indicators within the policy cycle and explicitly linking them to decision-making that accounts for climate-driven shifting baselines has been widely called for (*39*, *40*, *54*), yet no such framework currently exists for the Northeast Atlantic. Here, we advance this goal by developing a suite of complementary biodiversity indicators capable of assessing current status and projecting future trends across taxonomic groups in response to climate change.

## MATERIALS AND METHODS

### Survey data

Phytoplankton and zooplankton data were obtained from the Continuous Plankton Recorder survey (*55*, *56*). Samples were collected using a Continuous Plankton Recorder that is towed behind ships and continuously filters plankton from the water column using a moving band of silk gauze (270-μm mesh) at a depth of around 7 m. Observations with distinct spatial and temporal information correspond to a tow length of ∼10 nautical miles and a volume of 3 m^3^ of seawater (henceforth, plankton samples). Because it is unfeasible to count all phytoplankton in each sample, sample counts per taxa were derived by statistically resampling to estimate mean sample-level abundance. Phytoplankton counts were then rescaled to approximate the initial subsample counts (i.e., with many singletons that are typical of community samples and are required to estimate the effective number of species, see below) by dividing each taxa count by the lowest taxa count for that sample and rounded to the nearest whole number. Zooplankton counts were estimated at the sample-level using organisms ≥2 mm, meaning that no statistical resampling nor rescaling was required. We make use of phytoplankton (*n* taxa = 173) and zooplankton (*n* taxa = 119) data available from 1980 to 2021 for the northeast Atlantic downloaded on 9 July 2024, including information from 114,925 samples (fig. S9). Benthic macroinvertebrate data were sourced from the OneBenthic database (*57*) (https://rconnect.cefas.co.uk/onebenthic_portal/), with all publicly available data downloaded on 7 November 2024, including information on 3700 taxa from 29,403 comparable grab and core samples (henceforth, benthos samples, i.e., sampled using a 0.1-m^2^ grab or core and processed using a 1-mm sieve) collected between 1985 and 2023 from shelf waters of the northeast Atlantic (fig. S9). Fish observations from otter trawl surveys from across the northeast Atlantic spanning years 1983–2020 and including 513 taxa from 38,363 hauls were obtained from Lynam and Ribeiro (*58*) who collated multiple surveys stored on the ICES database of trawl surveys (fig. S9). Hauls (henceforth, fish samples) had a mean swept area of 0.7 km^2^ [standard deviation (SD) = 0.02; had a mean duration of 31.7 min].

All data on biota incorporate taxonomic information from the World Register of Marine Species (*59*), with each taxon uniquely identified by its “aphiaID,” allowing data to be outputted using standardized nomenclature. Taxonomic resolution varied considerably among taxonomic groups. Phytoplankton were identified with the lowest precision (15% to species, 46% to genus, none to family, and 39% only to class or higher). Most zooplankton records were resolved to species (69% to species, 6% to genus, 6% to family, and 19% to class or above), this precision was higher for benthic invertebrates (79% to species, 14% to genus, 3% to family, and 4% to class or higher). Fish achieved the highest resolution, with nearly all records (99%) identified to species and only 1% to genus or higher. Given this variability, we used the highest available taxonomic resolution for phytoplankton, zooplankton, and benthos, retaining records identified to species, genus, and family, while restricting fish to species-level data. This approach maximizes the retention of both taxonomic detail and abundance information across taxonomic groups, while acknowledging that diversity indices, particularly for phytoplankton, capture broader taxonomic richness rather than species richness alone.

### Diversity estimation

Rarefaction and extrapolation based on Hill numbers provide a unified framework for estimating α-, β-, and γ-diversity that corrects for biases arising from variable sample sizes and sampling effort and is applicable across different taxonomic groups (*31*, *32*, *60*). Diversity estimates were all generated per taxonomic group. To estimate α-diversity, we calculated species richness for each sample at twice the mean sample count for that taxonomic group (20 for phytoplankton, 42 for zooplankton, 365 for benthos, and 15,933 for fish) using individual-based rarefaction and extrapolation. γ-Diversity was estimated for the time and location of each sample by incorporating nine surrounding samples (selected at random where more than nine were available) collected within 6 months and within 75 km of that sample and applying sample-based rarefaction and extrapolation to 20 samples. Where <9 surrounding samples were available, γ-diversity estimates were dropped because variation in the number of samples can incorporate more or less β-diversity, biasing estimates (*61*). β-Diversity is an estimate of the effective number of communities in a given area and was calculated as

$$\beta =\gamma /\underline{\alpha }$$ (1)

where $\underline{\alpha }$ represents mean α-diversity (*60*, *62*).

α-, β-, and γ-diversity estimates were made for Hill number 0 (the effective number of species; i.e., species richness). These were supplemented with γ-diversity estimates made for Hill number 2 (the effective number of dominant species; i.e., inverse Simpson index). Moving from Hill number 0 to 2 progressively downweights the importance of rare species; hence, the contribution of rare species can be assessed by comparing across them. We assess the proportion of rare species as follows

$$1-\left(\gamma_{2}/\gamma_{0}\right)$$ (2)

where γ_2_ represents γ-diversity of Hill number 2 (dominant species) and γ_0_ represents γ-diversity of Hill number 0 (all species). Because the BART models for γ_0_ and γ_2_ were fitted separately, they do not enforce the mathematical constraint γ_0_ ≥ γ_2_ everywhere, which can occasionally yield implausible rare-species fractions slightly outside the admissible interval [0,1]. These artifacts were extremely uncommon and very small in magnitude: Out-of-range values were almost exclusively minor negative deviations close to zero (median, −0.02 to −0.11), and, on average, only 0.00 to 0.08% of grid cells per taxonomic group and scenario showed any out-of-range values (maximum, 0.07 to 0.70%; table S1 and fig. S10). All values were clipped to [0,1] before computing temporal averages and rates of change. Given their rarity and minimal size, these corrections have no influence on the results. All analyses for the study were conducted using the R Statistical software (*63*). Rarefaction and extrapolation analyses were performed using the R package iNEXT (*64*).

### Environmental predictors

Our models incorporated two classes of environmental predictors: dynamic variables that varied annually and static variables that remained constant through time.

1) Dynamic variables. Statistically downscaled CMIP6 monthly projections of temperature, salinity, and oxygen for the time period 1993–2100 (*65*) provided temporally varying environmental data. This 1/12-degree downscaled dataset was regridded onto a 5 km–by–5 km grid, and annual averages were calculated. For each grid cell and each year, the SDs of all monthly values of temperature, oxygen, and salinity within a 75-km spatial radius were also calculated to include intra-annual environmental heterogeneity of the physical variables within the model known to be important in marine ecosystem diversity assessments (*47*, *61*).

2) Static variables. The static variables used were bathymetry (from the General Bathymetric Chart of the Oceans; www.gebco.net, at 15-s resolution), substrate composition (*66*) (sand, mud and gravel percentages), and shear stress (*67*, *68*) (calculated as the mean of the annual averages in the 7-year period 1975–1981 and the SD of those annual averages). These data were regridded onto the same 5 km–by–5 km grid. The distance from each grid-cell center to the nearest coastline was also estimated.

Analyses for phytoplankton and zooplankton used sea surface environmental variables only, whereas analyses for benthic organisms and fish used seabed estimates of the dynamic variables together with additional static seafloor variables (e.g., shear stress, its SD, and sediment composition). Samples were linked to the environmental data using their date and spatial information. Where there were multiple samples for a taxonomic group per grid cell per year, mean estimates were calculated for each of the biodiversity indices. The final number of grid cells with diversity estimates were 23,345, 25,321, 8505, and 23,320 for phytoplankton, zooplankton, benthos, and fish, respectively.

### Selection of environmental predictors and biodiversity indices

If an environmental variable was highly correlated with another (*R*^2^ > 0.7), then the less ecologically relevant one was excluded. Because sample locations differed among taxonomic groups, correlations between environmental predictors also varied, resulting in group-specific sets of explanatory variables (fig. S8). After removing highly correlated predictors, the variables included in the BART models for phytoplankton and zooplankton were sea surface temperature and salinity, bathymetry, and distance to coast; for benthos, sea bottom oxygen and its SD, sea bottom salinity and temperature, shear stress and its SD, mud and sand percentages, bathymetry, and distance to coast; and for fish, all static and dynamic environmental variables were retained.

### Statistical modeling

We used BART (*69*) to project the biodiversity indices in time and space for each of the four taxonomic groups based on their relationships with the environmental predictors. BART is a machine learning approach that combines an ensemble of regression trees, with each tree estimating a small and different part of the response variable. Predictions are made through sequential splitting of explanatory variables. Unlike other ensemble tree methods, such as Random Forest and Boosted Trees, BART constructs its ensemble within a Bayesian framework, where both tree structure and terminal node predictions are governed by prior distributions. Similar to other tree-based approaches, not only it can represent nonlinear relationships and interactions among variables and handle missing data, but it also provides formal uncertainty estimates—a feature often missing from other spatial distribution models—while performing as well as or better than comparable methods (*69*, *70*). We used the “bartMachine” R package (*71*) for our modeling.

The BART models were trained in the periods 1993–2021 (phytoplankton and zooplankton), 1993–2023 (benthos), and 1993–2020 (fish) and then projected over the wider region (Fig. 1) for the period 1993–2100. We projected biodiversity change under three climate change scenarios representing low, medium, and high greenhouse gas emissions (SSP1-2.6, SSP2-4.5, and SSP5-8.5). Here, we primarily focus on SSP2-4.5 to explore spatial patterns of change, with results for other scenarios provided in figs. S1 to S5. The prediction region used for plankton is larger than for benthos and fish (Fig. 1) because the substrate composition data used for the latter models is spatially limited and additionally because the benthic and fish sample data are limited to the coastal shelf, so we avoid projecting far from the model training region.

To assess model performance, BART models were evaluated using 10-fold cross-validation. The training data were randomly partitioned into 10 subsets. For each fold, the model was trained on nine subsets and tested on the remaining one. Performance was assessed by calculating the pseudo-*R*^2^ between predicted and observed values. The validity of model predictions were also assessed using MESS maps (*72*). MESS maps identify regions and time periods where environmental explanatory variables are out of range of that used to train the model and thus where predictions are considered less reliable. For the maps in Figs. 1 and 2, hashed out areas indicate regions where any environmental variable is outside the training range (MESS values < 0). A summary of the modeling steps used in this study is given in Fig. 4.

**Fig. 4.**
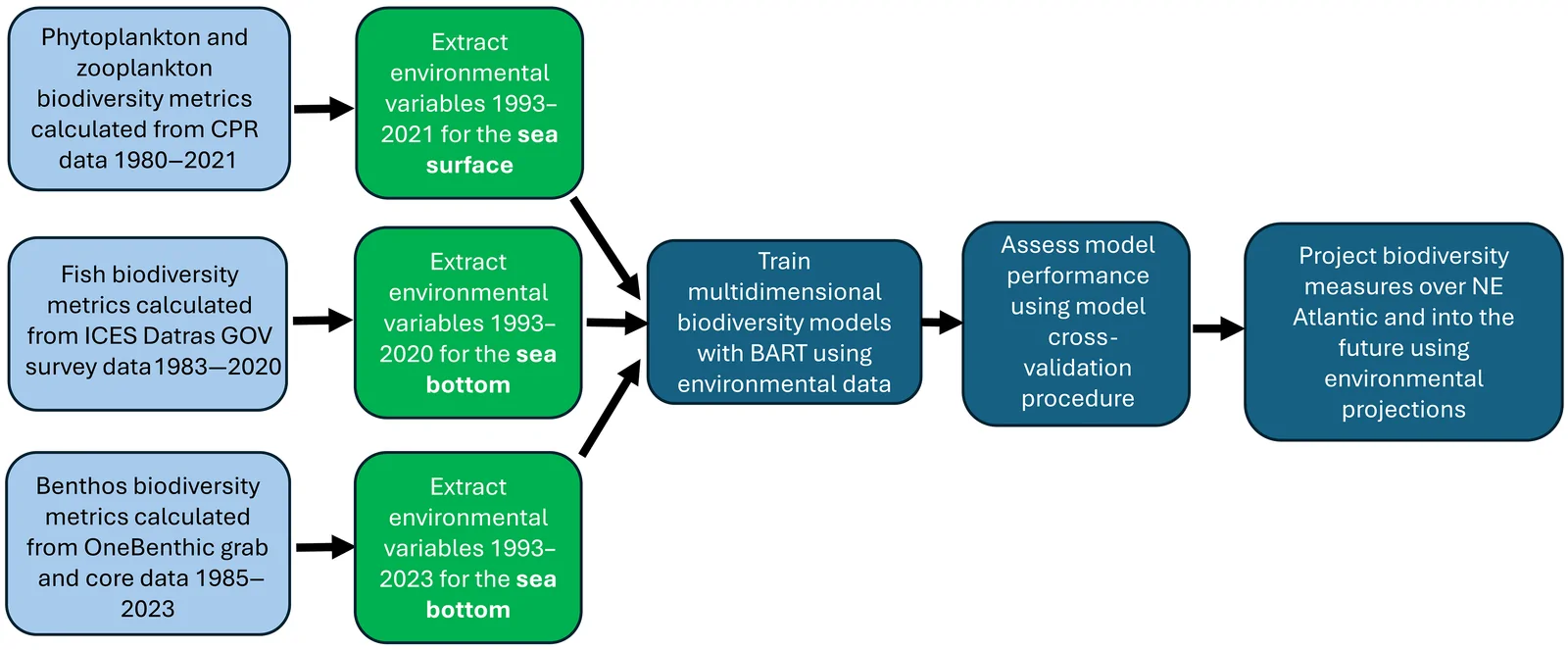
Study workflow. Workflow diagram summarizing the sequential steps of the study, from diversity estimations for the four taxonomic groups to future projections.

Sen’s slope is a nonparametric method that can quantitatively estimate the magnitude of change where assumptions for linear regression are not met (*73*), and we use this to capture change over time in our biodiversity indices. For each grid cell, we compute Sen’s slope for the linear rate of change between each biodiversity index and year between 1993 and 2100 based on the different emission scenarios and report whether temporal trends were significant using the trend R package (*74*).

We integrate the diversity across the four taxonomic groups by calculating the value of total sum of γ-diversity to investigate overall biodiversity patterns. To do so, we normalized the γ-diversity for each taxonomic group to a scale of 0 to 1 and then summed across them. The final values range between 0 and 4 and represent the total sum of γ-diversity. Change from the total sum of γ-diversity was then estimated for different periods using the reference period (2000–2019) as the baseline.

In Fig. 5, we illustrate the processes driving temporal change in spatial biodiversity patterns. Regional species richness increases when the arrival of new species outpaces extirpations (*75*). If newcomers are rare (i.e., with low spatial occupancy), both species richness and the percentage of rarity will increase. In contrast, if arrivals are mainly abundant species (i.e., rapidly becoming widespread), then species richness will increase, but the percentage of rarity will decrease. Changes can also occur through shifts in the occupancy of existing species with no change in species richness: range contraction of abundant species increases rarity, while range expansion of rare species decreases rarity. Where regional extirpation exceeds regional species increases: If losses are skewed toward rare species, then both species richness and rarity decline, whereas if abundant species are mostly lost, then richness decreases but the proportion of rarity increases. These processes are not mutually exclusive, meaning when temporal change is observed, multiple mechanisms may be acting simultaneously to shape patterns of biodiversity. We aimed to reveal the dominant processes determining temporal change in spatial biodiversity patterns and how changing environmental conditions could affect these.

**Fig. 5.**
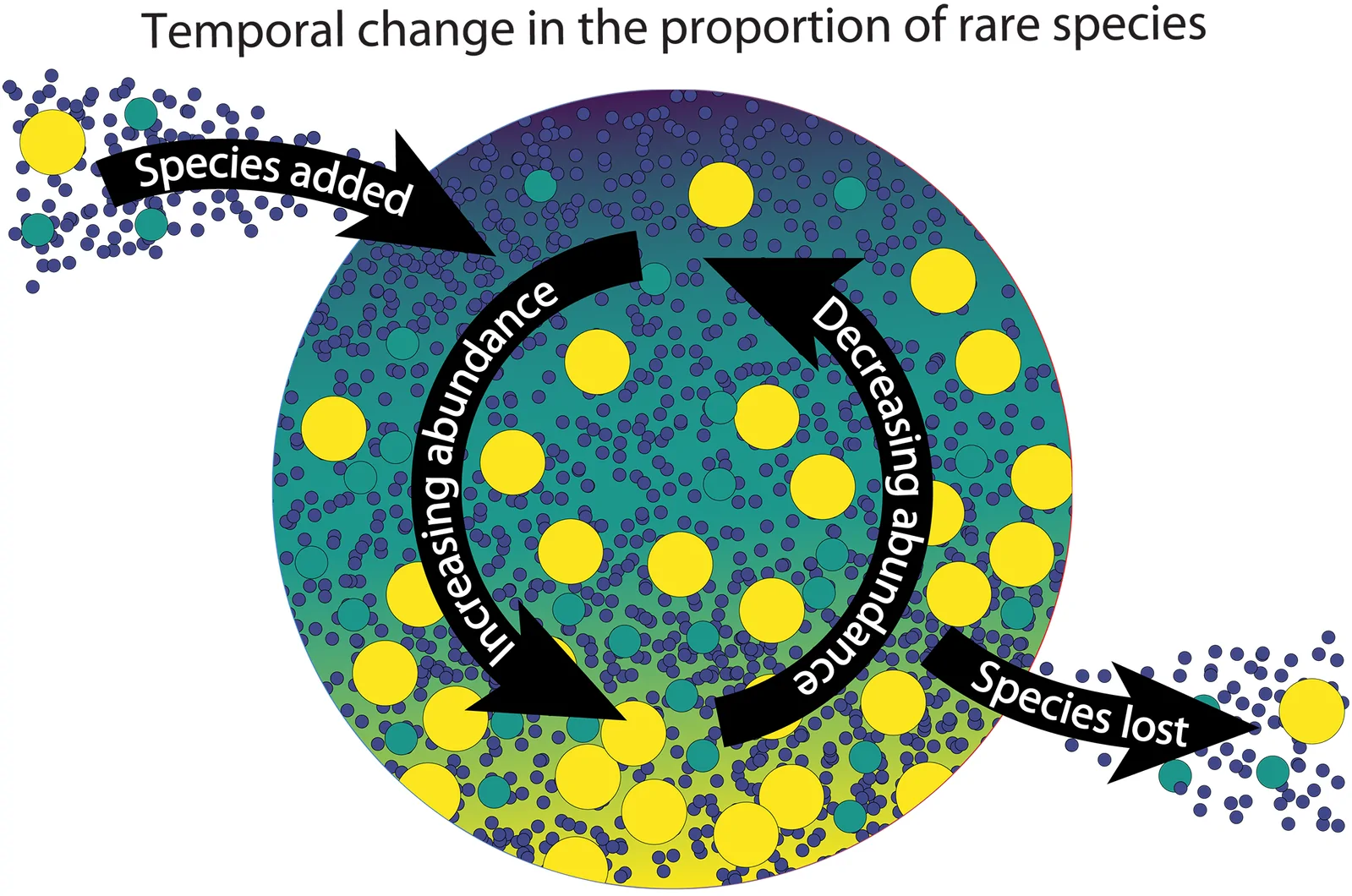
Processes driving rare-species change. Changes in the proportion of rare species within a region can result from three processes: the arrival of new species, the disappearance of existing species, and shifts in the abundance/range of species already present.
