# Supplementary material for: From plankton to fish: 21st-century redistribution of marine biodiversity and the changing role of rare species
Source: Sci Adv. 2026 Oct 7;12(41):eaee7253. doi: 10.1126/sciadv.aee7253 (PMC13644749; doi:10.1126/sciadv.aee7253)
Supplement: Supplementary file 1 [file sciadv.aee7253_sm.pdf]

Science Advances
AAAS

Supplementary Materials for

From plankton to fish: 21st-century redistribution of marine biodiversity and the changing role of rare species

Elena Couce *et al.*

Corresponding author: Elena Couce, elena.couce@cefas.gov.uk

Sci. Adv. **12**, eaee7253 (2026)
DOI: 10.1126/sciadv.aee7253

This PDF file includes:

Figs. S1 to S10
Table S1

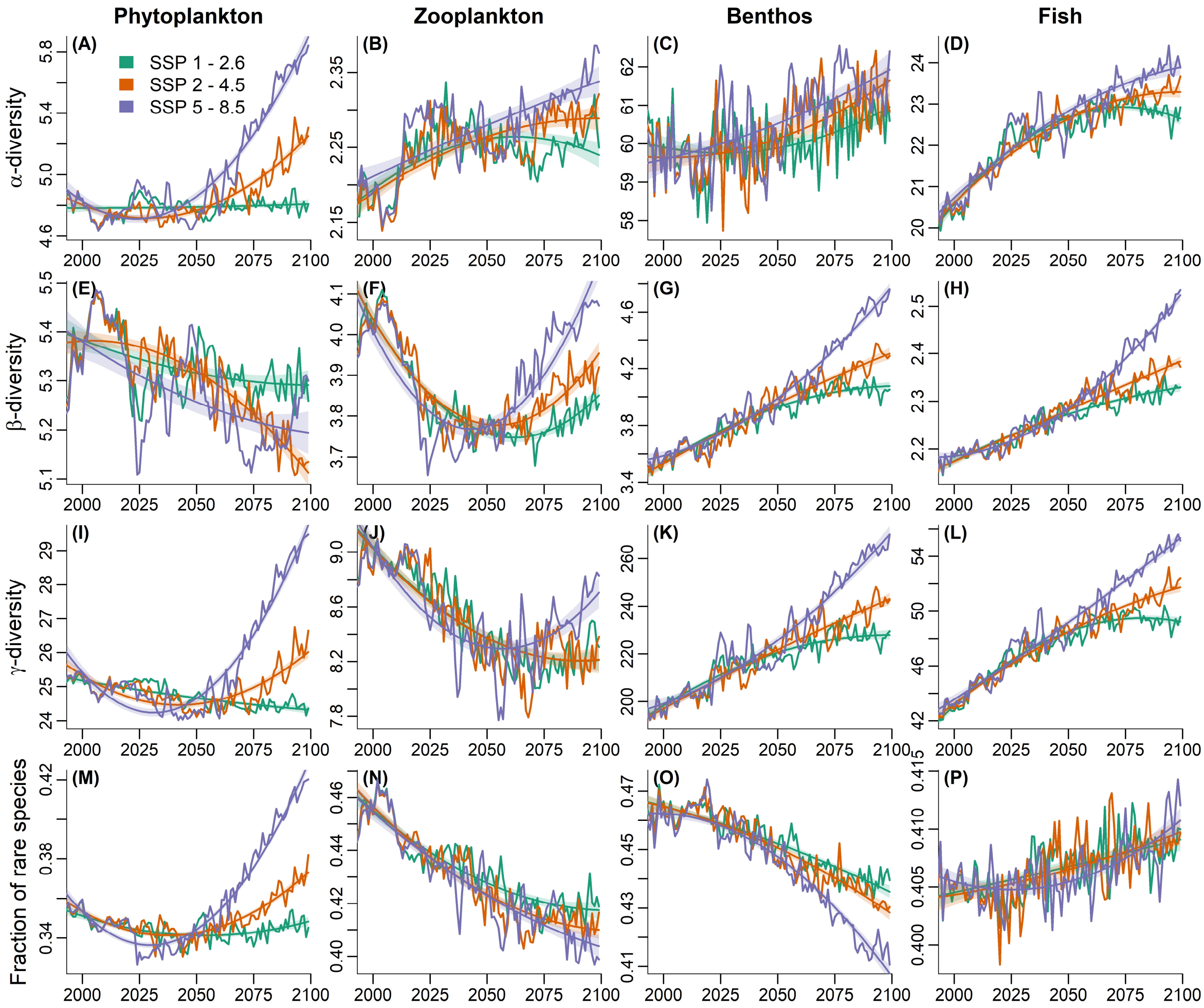

Fig. S1. Projected diversity trends across SSPs. Projected mean temporal trend across the study region in α- (**A-D**), β- (**E-H**), γ-diversity (**I-L**), and the proportion of rarer species (**M-P**) for phytoplankton (**A,E,I,M**), zooplankton (**B,F,J,N**), benthos (**C,G,K,O**) and fish (**D,H,L,P**) for the three emission scenarios SSP1-2.6, SSP2-4.5 and SSP5-8.5.

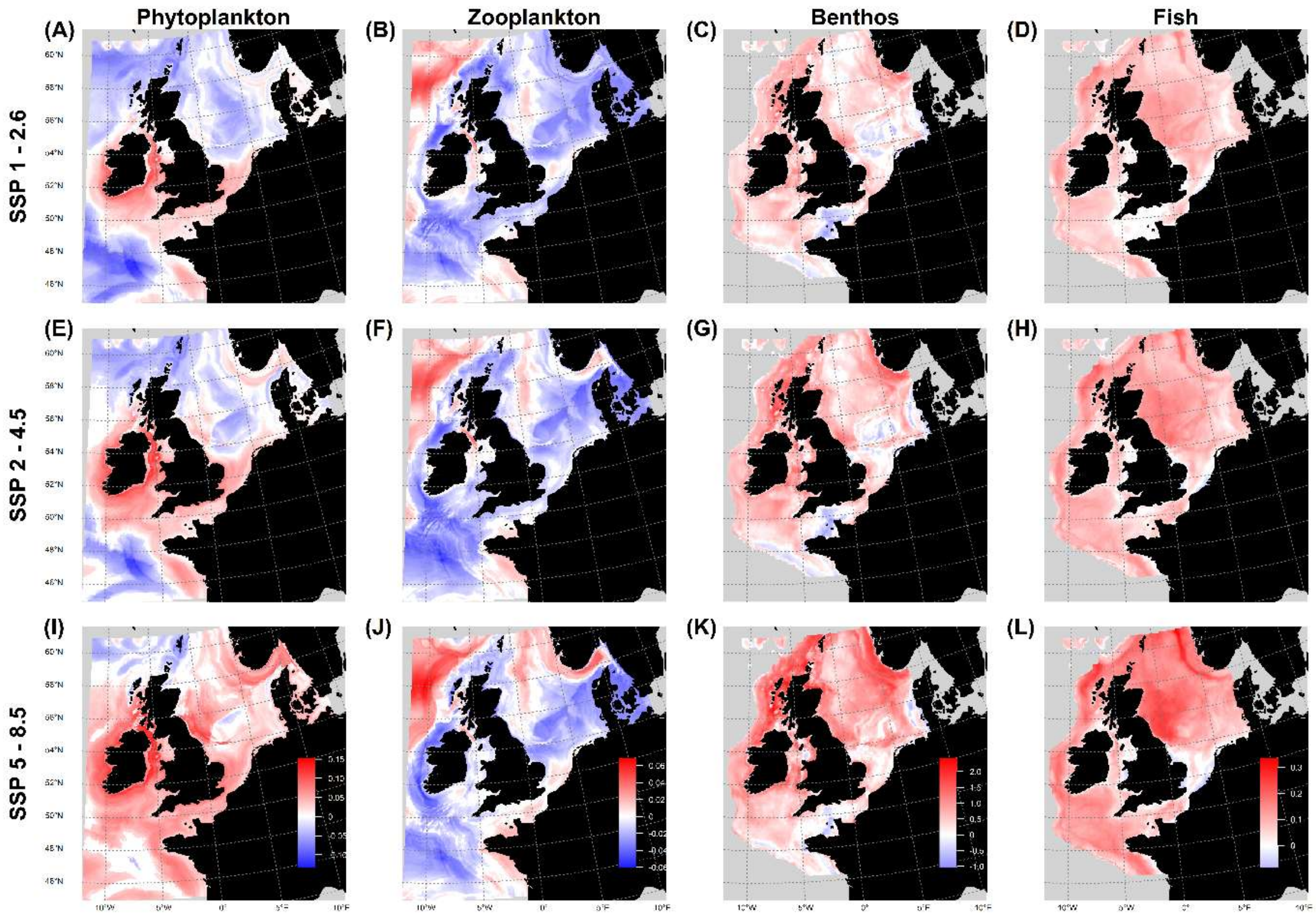

Fig. S2: Projected trends in γ-diversity. The projected annual change (Sen's slope) in γ-diversity (Hill number 0) for phytoplankton (**A,E,I**), zooplankton (**B,F,J**), benthos (**C,G,K**), and fish (**D,H,L**) from 1993 – 2100 based on SSP1-2.6 (**A-C**), SSP2-4.5 (**E-H**) and SSP5-8.5 (**I-L**). The colourbars shown in the bottom row (plots **I-L**) apply to each column. Red and blue cells show significant ($p < 0.05$) increasing and decreasing annual change, respectively, with white cells showing areas with non-significant change.

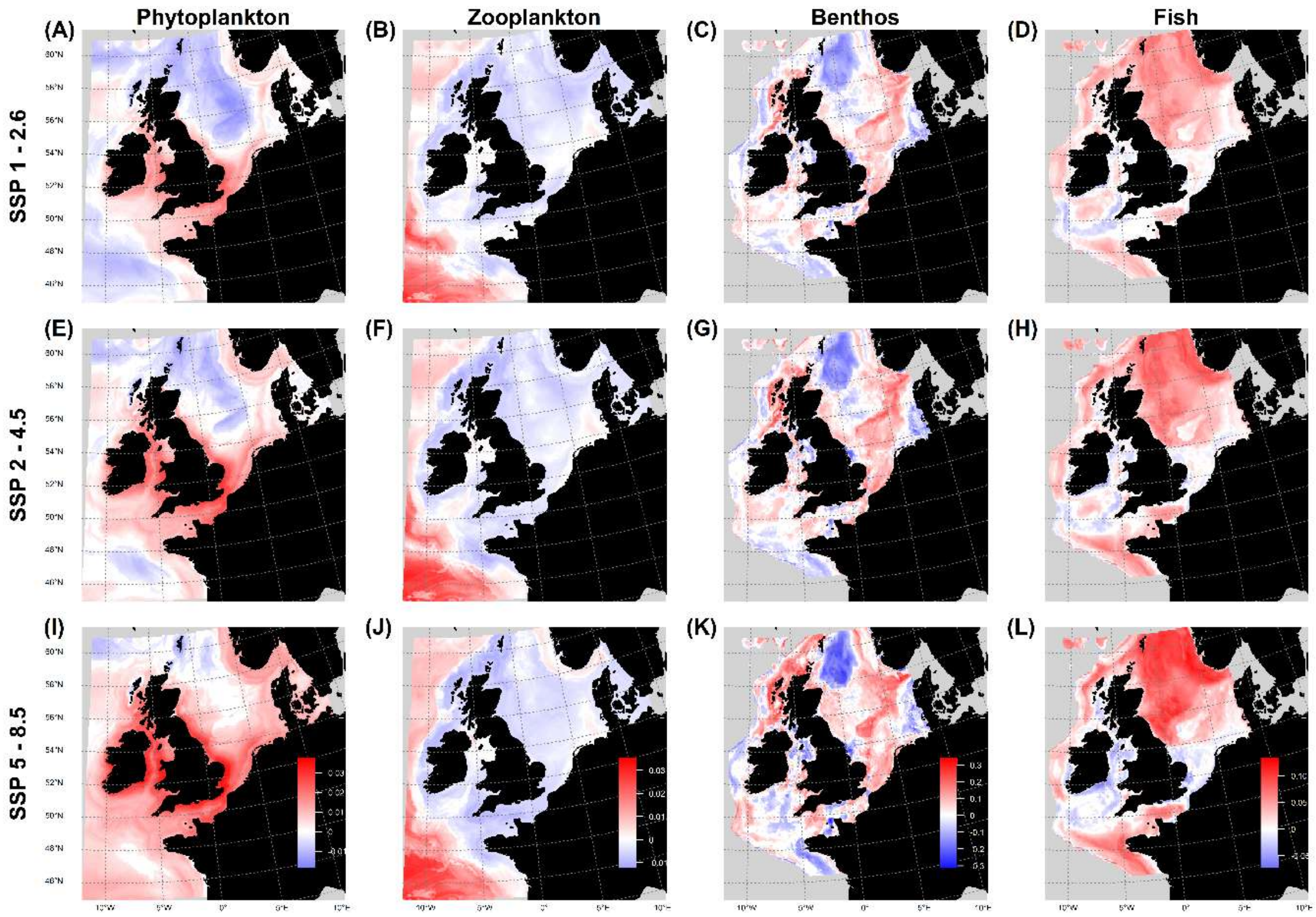

Fig. S3. Projected trends in α-diversity. The projected annual change (Sen's slope) in α-diversity (Hill number 0) for phytoplankton (**A,E,I**), zooplankton (**B,F,J**), benthos (**C,G,K**), and fish (**D,H,L**) from 1993 – 2100 based on SSP1-2.6 (**A-C**), SSP2-4.5 (**E-H**) and SSP5-8.5 (**I-L**). The colourbars shown in the bottom row (plots **I-L**) apply to each column. Red and blue cells show significant ($p < 0.05$) increasing and decreasing annual change, respectively, with white cells showing areas with non-significant change.

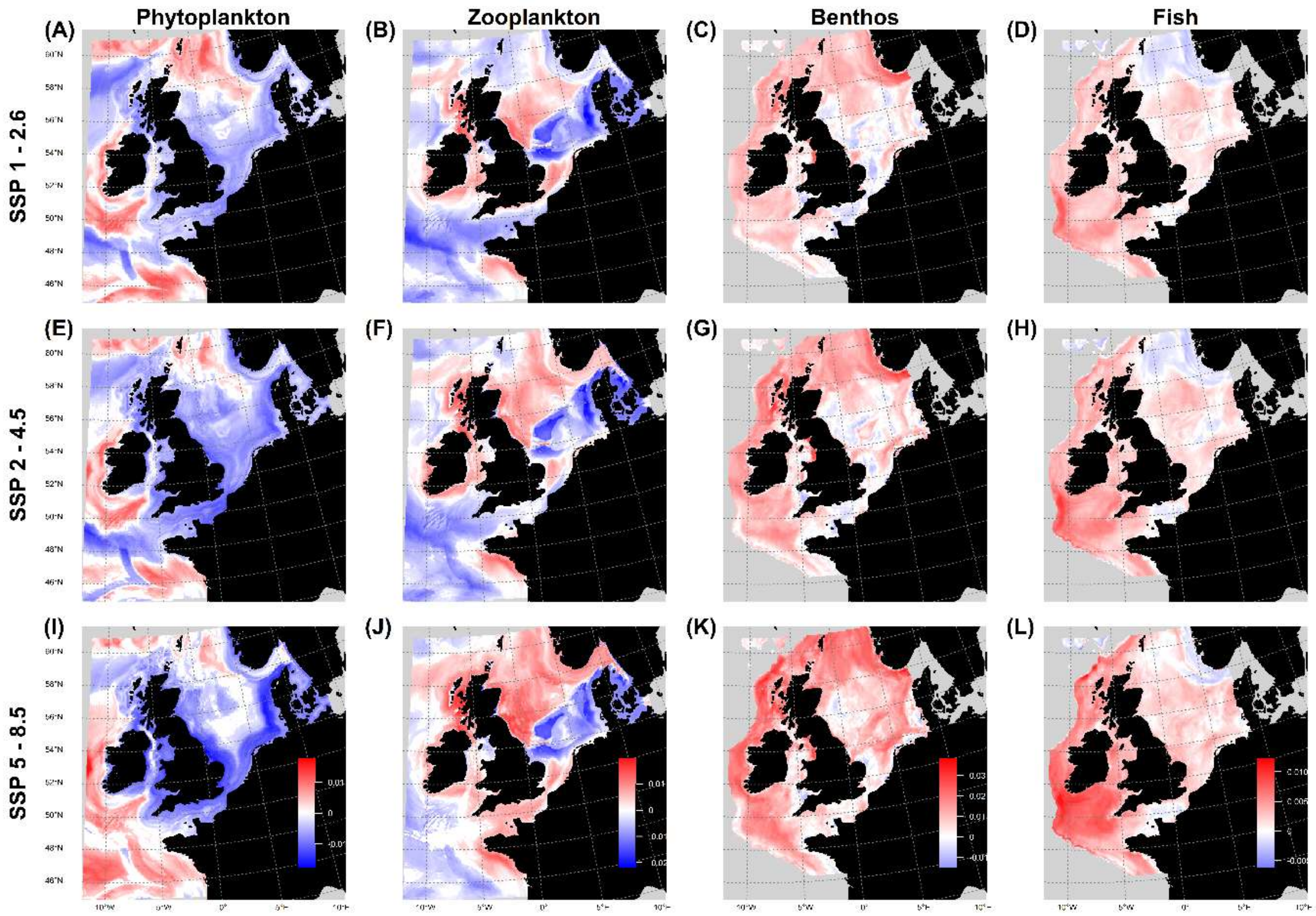

Fig. S4. Projected trends in β-diversity. The projected annual change (Sen's slope) in β-diversity (Hill number 0) for phytoplankton (**A,E,I**), zooplankton (**B,F,J**), benthos (**C,G,K**), and fish (**D,H,L**) from 1993 – 2100 based on SSP1-2.6 (**A-C**), SSP2-4.5 (**E-H**) and SSP5-8.5 (**I-L**). The colourbars shown in the bottom row (plots **I-L**) apply to each column. Red and blue cells show significant ($p < 0.05$) increasing and decreasing annual change, respectively, with white cells showing areas with non-significant change.

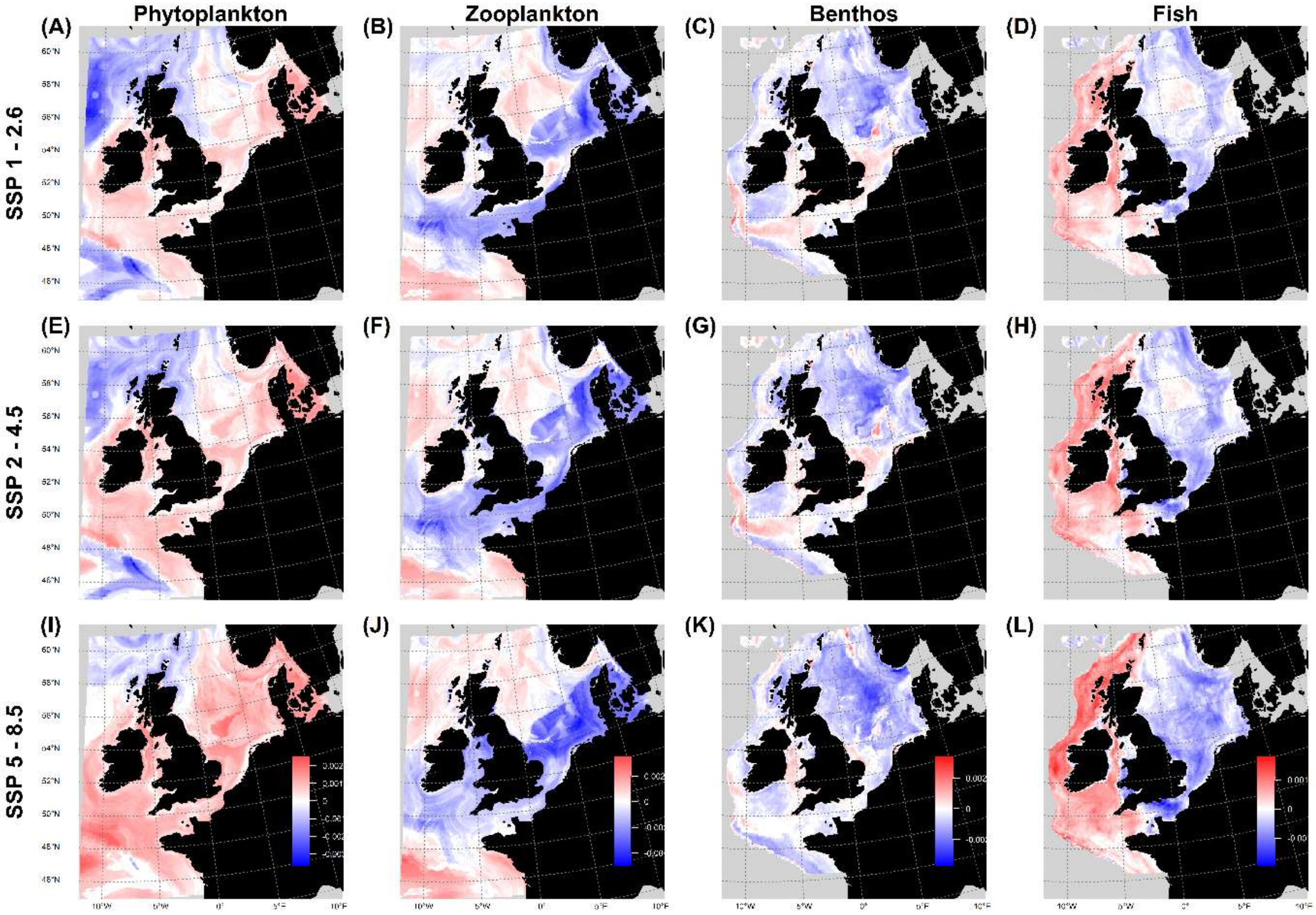

Fig. S5. Projected trends in proportion of rare species. The projected annual change (Sen's slope) in the fraction of rare species for phytoplankton (**A,E,I**), zooplankton (**B,F,J**), benthos (**C,G,K**), and fish (**D,H,L**) from 1993 – 2100 based on SSP1-2.6 (**A-C**), SSP2-4.5 (**E-H**) and SSP5-8.5 (**I-L**). The colourbars shown in the bottom row (plots **I-L**) apply to each column. Red and blue cells show significant ($p < 0.05$) increasing and decreasing annual change, respectively, with white cells showing areas with non-significant change.

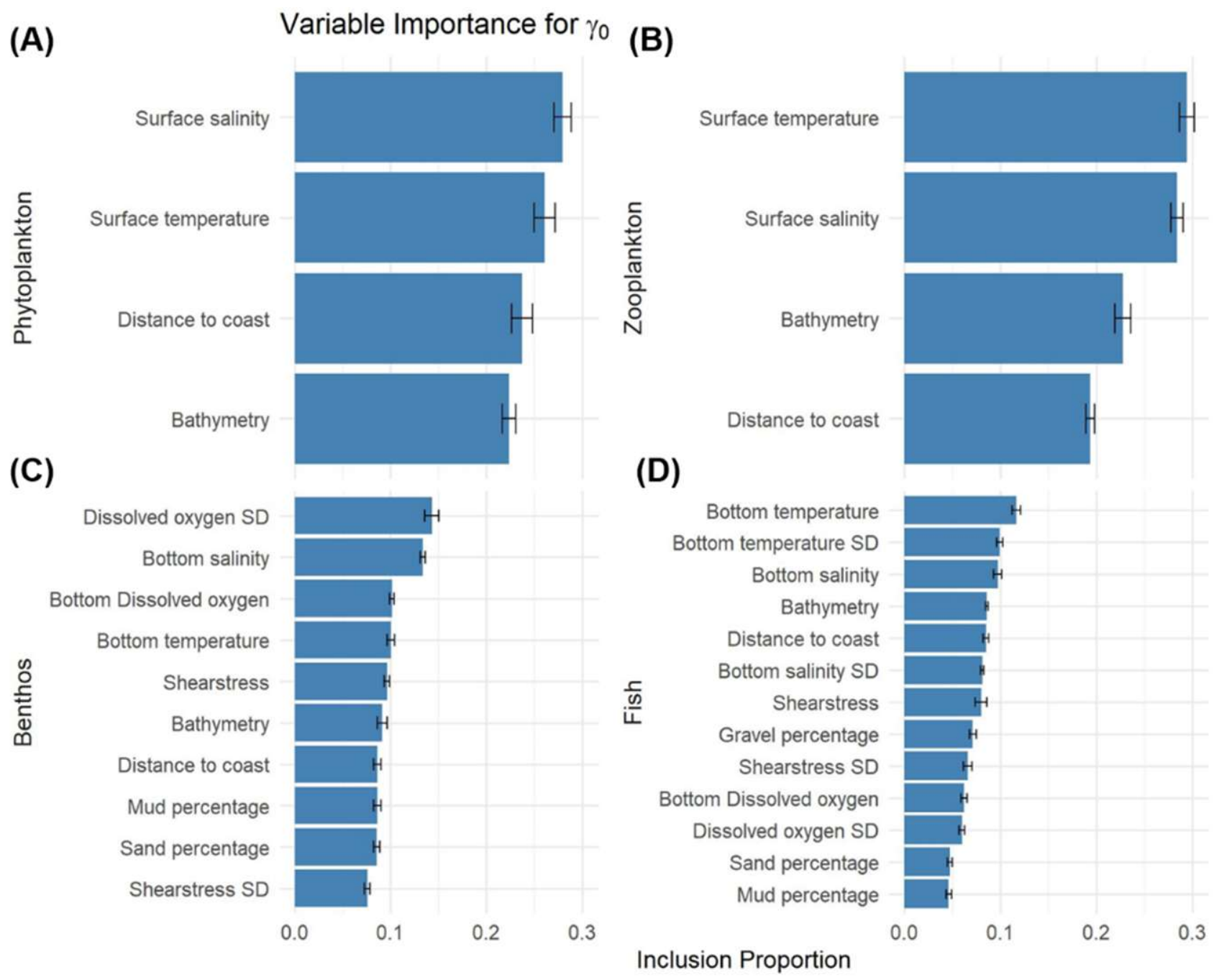

Fig. S6. γ-diversity (Hill 0) variable importance. Variable importance for the models of γ-diversity based on Hill number 0 for (**A**) phytoplankton, (**B**) zooplankton, (**C**) benthos, and (**D**) fish.

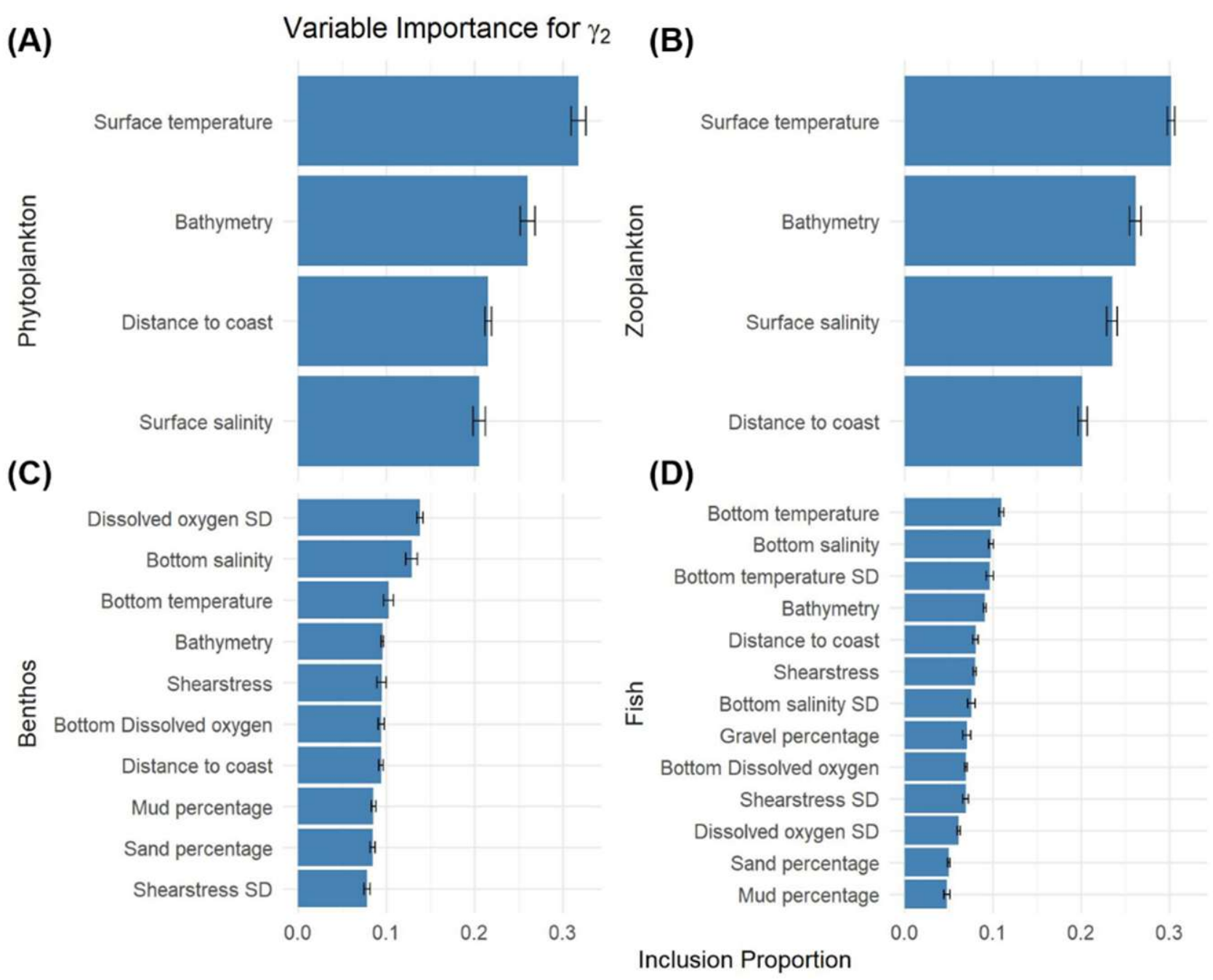

Fig. S7. γ-diversity (Hill 2) variable importance. Variable importance for the BART models of γ-diversity based on Hill number 2 for (**A**) phytoplankton, (**B**) zooplankton, (**C**) benthos, and (**D**) fish.

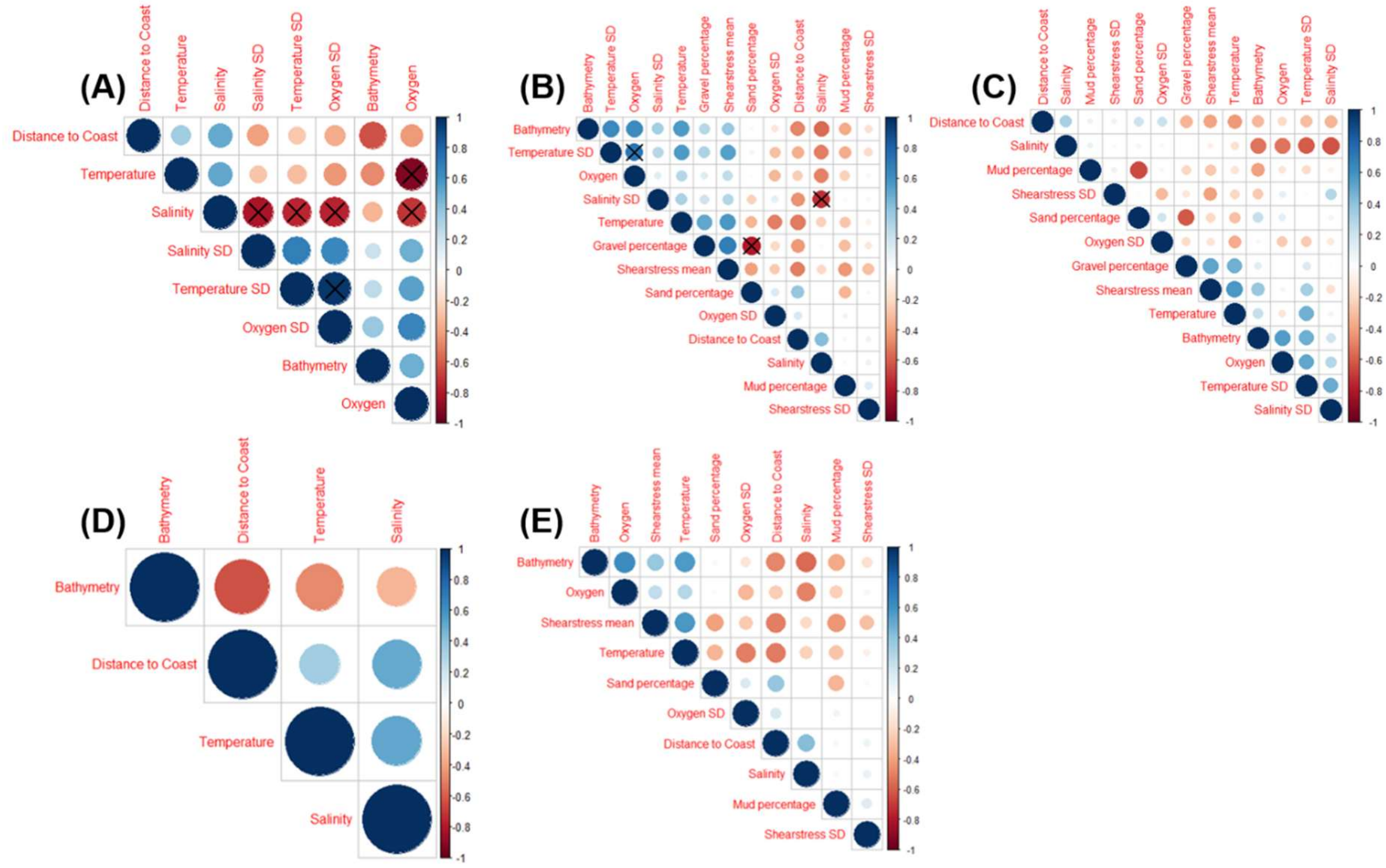

Fig. S8. Pairwise correlations among environmental variables. Pairwise Pearson correlation coefficients among all environmental covariates, evaluated based on available observations. Black crosses indicate correlations exceeding 0.7 across the full set of environmental variables used for (**A**) phytoplankton and zooplankton, (**B**) benthos, and (**C**) fish. The final set of variables included in our models for (**D**) phytoplankton and zooplankton and (**E**) benthos, respectively. All variables were retained for fish. The suffix “SD” denotes the standard deviation of the 12 monthly means per year across all locations within a 75 km radius of each grid cell, as a measure of environmental spatio-temporal heterogeneity. For shear stress, “SD” refers instead to the standard deviation across 7 annual means, because data were not available at the same spatio-temporal resolution as the other variables.

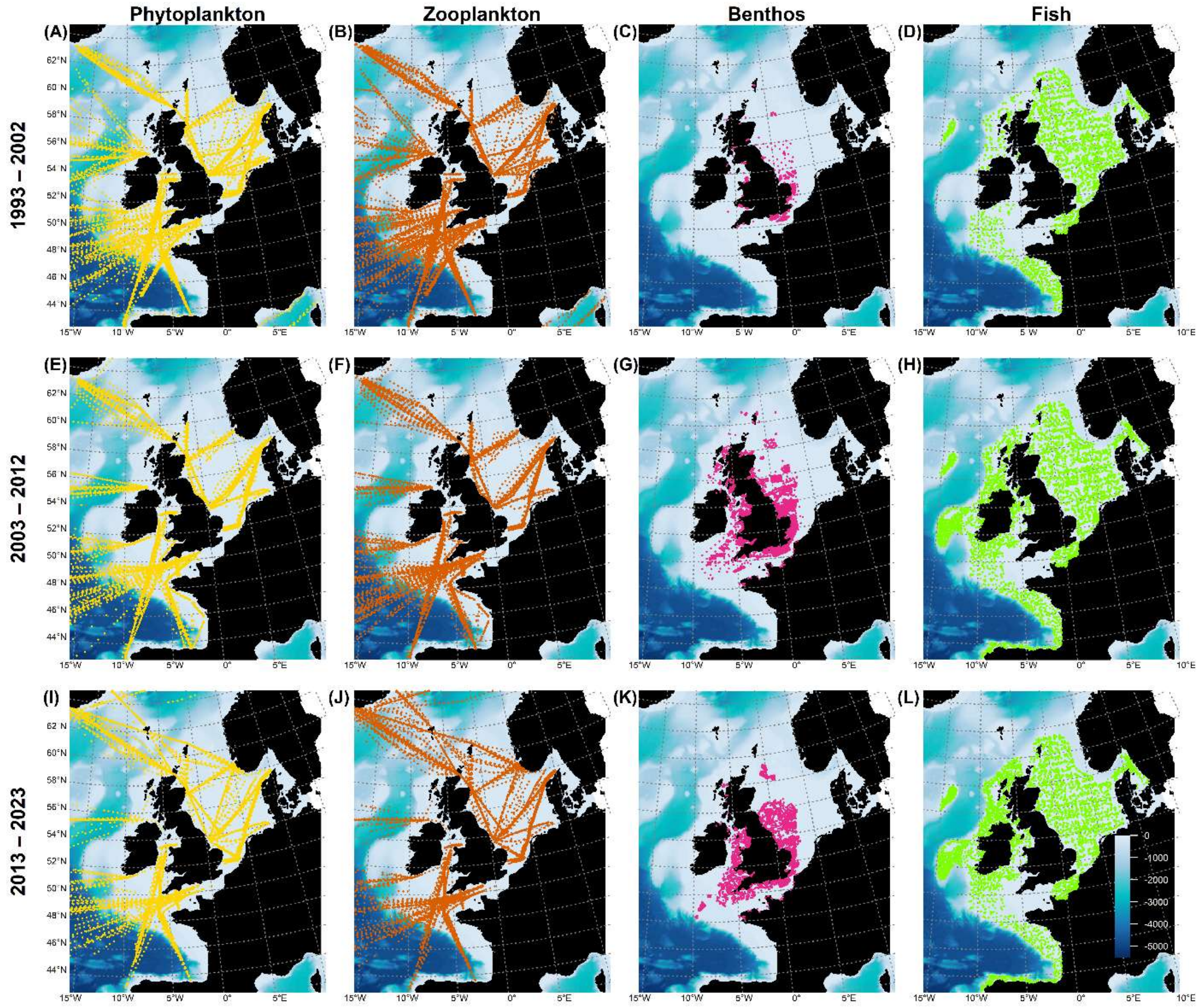

Fig. S9. Decadal survey coverage across taxonomic groups. Spatial distribution of sampling points for 1993–2002 (**A-D**), 2003–2012 (**E-H**), and 2013–2023 (**I-L**), shown over a bathymetric colour map (blue). Phytoplankton (**A,E,I**) and zooplankton (**B,F,J**) samples exhibit broadly similar spatial coverage, benthic samples (**C,G,K**) are concentrated along the coast, and fish samples (**D,H,L**) are largely restricted to the continental shelf.

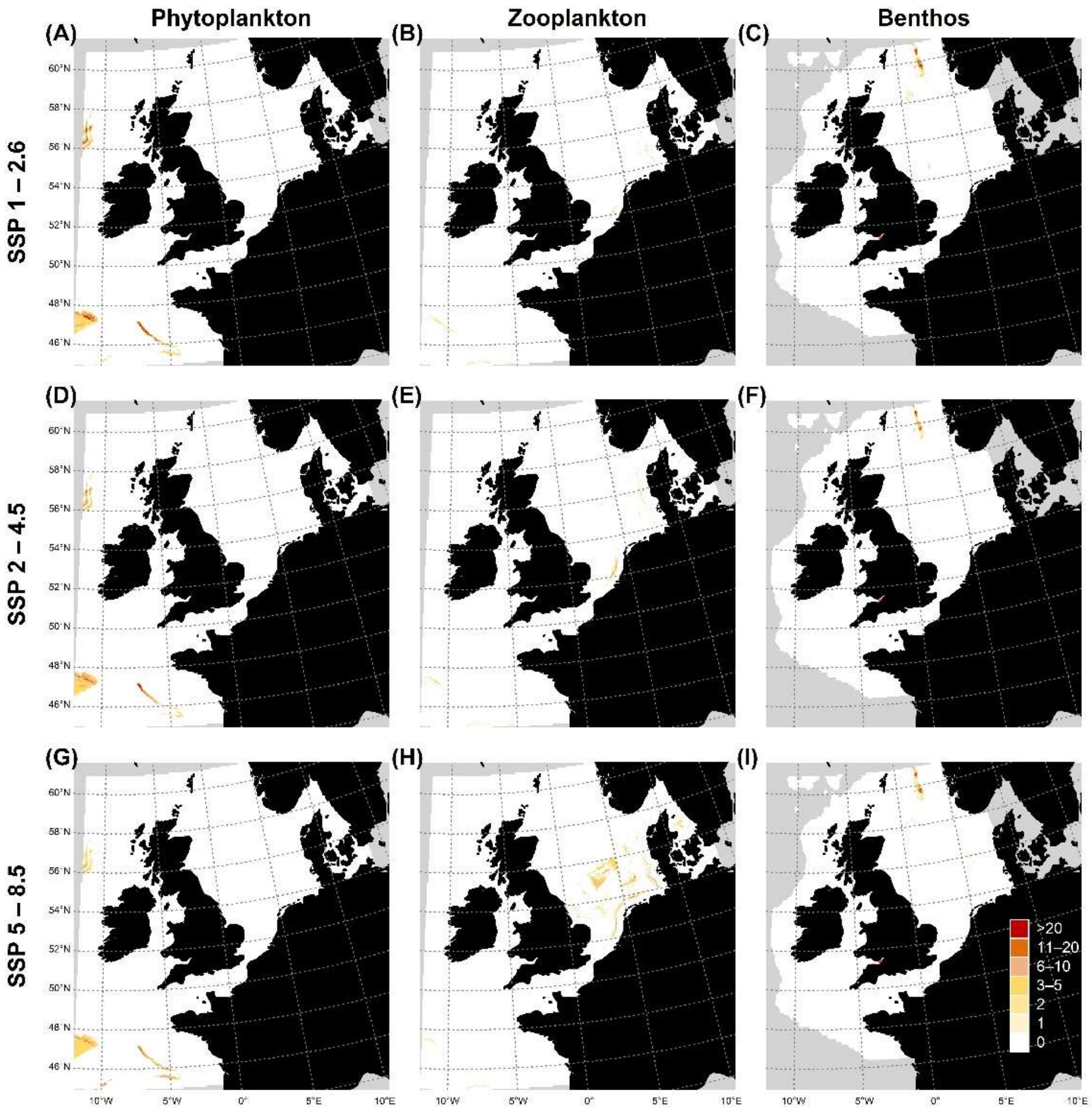

Fig. S10. Spatial distribution of out-of-range predictions. Spatial distribution of years with out-of-range rare-species predictions. For each taxonomic group and emissions scenario, panels show the number of years in 1993–2099 in which predicted rare-species fractions fell outside the admissible interval [0,1]. Although these occurrences are rare overall (Table S1), they display some geographic structure, with clusters of affected cells emerging in particular regions for some taxonomic groups and scenarios. Fish are not shown because no out-of-range values occurred in any year or scenario. All out-of-range values arise from minor numerical artefacts associated with independently modelled diversity components, and were clipped to [0,1] prior to analysis (Methods).

Table S1. Prevalence and magnitude of out-of-range predictions of the rare-species fraction. For each taxonomic group and emissions scenario, the table reports the mean and maximum percentage of grid cells, and the mean and maximum number of cells (n), in which predicted rare-species fractions fell outside [0,1] during 1993–2099. Out-of-range predictions were extremely uncommon (typically <0.1% of grid cells) and mostly involved small deviations close to zero (Methods). These artefacts were corrected by clipping values to [0,1] prior to subsequent analyses.

Taxonomic group	SSP	Mean % clipped	Max % clipped	Mean n clipped	Max n clipped
Benthos	SSP1-2.6	0.045	0.205	20.38	93
	SSP2-4.5	0.032	0.157	14.65	71
	SSP5-8.5	0.037	0.273	16.88	124
Fish	SSP1-2.6	0.000	0.000	0.00	0
	SSP2-4.5	0.000	0.000	0.00	0
	SSP5-8.5	0.000	0.000	0.00	0
Phytoplankton	SSP1-2.6	0.078	0.616	52.01	411
	SSP2-4.5	0.065	0.685	43.28	457
	SSP5-8.5	0.055	0.701	36.84	468
Zooplankton	SSP1-2.6	0.006	0.147	3.93	98
	SSP2-4.5	0.010	0.070	6.77	47
	SSP5-8.5	0.053	0.493	35.61	329
